# Supplementary material for: Are carbon emissions trading and green financial instruments synergistic? -Comprehensive quantitative research based on content analysis
Source: PLoS One. 2024 Mar 7;19(3):e0298601. doi: 10.1371/journal.pone.0298601 (PMC10919601; doi:10.1371/journal.pone.0298601)
Supplement: S1 Data — (ZIP) [file pone.0298601.s001.zip › Data/地级市Z_F工作报告.zip!/╣π╢1⁄2/╓╨╔╜╩╨/└┤╫╘╣1⁄2╓┌║┼í╛┬φ┐╦╩2╛▌═°í┐_2017.docx]

各位代表：

　　我代表市人民政府向大会作政府工作报告，请予审议，并请政协委员和其他列席人员提出意见。

　　过去五年工作回顾

　　过去五年，是我们积极应对严峻复杂形势，全市上下众志成城取得重要成就的五年。五年来，市政府在省委、省政府和市委的正确领导下，在市人大及其常委会和市政协的监督支持下，全面贯彻党的十八大和十八届三中、四中、五中、六中全会精神，深入贯彻习近平总书记系列重要讲话精神，坚持稳中求进工作总基调，积极适应和主动引领经济发展新常态，深入推进供给侧结构性改革，大力实施创新驱动发展战略，统筹做好稳增长、促改革、调结构、惠民生、防风险各项工作，有效应对经济下行压力，经济社会发展取得新进步。

　　过去五年，是经济实力不断增强的五年。2016年，全市生产总值3202.8亿元，比2011年增加超千亿，年均增长9.1%。人均生产总值9.95万元，是2011年的1.4倍，居珠三角前列。工业增加值1606.2亿元，服务业增加值1457.3亿元，分别是2011年的1.4倍和1.6倍。民营经济增加值占生产总值比重达52.9%，比2011年提高6.9个百分点。固定资产投资1149亿元，五年累计近5000亿元。社会消费品零售总额1205.8亿元，是2011年的1.6倍，年均增长10.5%。外贸进出口2237.7亿元，增长1.3%。一般公共预算收入295亿元，是2011年的1.6倍，年均增长9.9%。城镇化率提高到88.15%。

　　过去五年，是结构调整深入推进的五年。翠亨新区上升为省级战略发展平台。三次产业结构从2011年的2.7:55.8:41.5 优化为2.2:52.3:45.5。农业发展更加高效，休闲农业、观光农业等农业新业态快速发展，五年来建设高标准基本农田17.8万亩。工业高端化、智能化趋势更加明显，五年来先进制造业、高技术制造业增加值占规上工业增加值比重分别提高8.3和6 个百分点。骨干企业成长加快，亿级企业达1188家，十亿级企业超100家。规上工业全员劳动生产率达16.5万元/人。服务业支撑作用更加突出，五年来服务业增加值占生产总值比重提高4个百分点。现代服务业占服务业增加值比重达61.1%。金融活力更加强劲，境内外上市挂牌企业从2011年的15家增至81家，建成众创金融街。投资结构更加优化，工业技改投资达 205.5 亿元，是2011年的2.5倍。民间投资占比从2011 年的59.1%提高到69.4%。出口产品更具竞争力，机电产品出口占比达70.7%，五年来一般贸易出口占比提高10个百分点。

　　过去五年，是创新能力稳步提升的五年。成为全国科技进步考核先进市和国家知识产权示范市。在2015年度创新驱动发展考核中居全省第4。珠三角国家自主创新示范区建设加快推进，火炬区“一区多园”模式启动实施，中德（中山）生物医药产业园成为国家重点对德合作园区。高新技术企业从2011年的236家增至884家，近两年增速分别居全省第1和第2。科技孵化器增至48家，其中国家和省级15家。市级以上新型研发机构增至46家。发明专利申请量和授权量分别是2011年的5.6倍和3.1倍。全社会研发经费占生产总值比重提高到2.6%，居全省前列。人才创新生态不断完善，五年来培育引进各类人才27万人，入选中央“千人计划”8人。成功创建国家级留学人员创业园，博士后工作平台增至42家，创新创业基地增至26家，省市创新创业团队增至27个。在中国创新创业大赛广东赛区中获奖数居全省第2。

　　过去五年，是改革开放全面深化的五年。供给侧结构性改革取得重大进展，2016年淘汰印染行业落后产能3500万米，减少商品房库存面积200多万平方米，降低企业各类成本约92亿元。市属国有企业资产证券化取得突破，3家市属国有企业实现新三板挂牌。建成市行政服务中心和网上办事大厅，推行审批职能“三集中一分开”改革。推进“一门式、一网式”政府服务模式改革，建成市、镇、村三级多功能综合服务平台。全面取消非行政许可审批事项，行政审批中介服务费用和时限分别压缩20%和50%。成为全省首批企业投资项目清单管理试点市。简政强镇事权改革有序推进，五年来下放镇区1793项行政审批和执法事项。解决南区、西区“一区两制”问题。深化商事制度改革，实施“五证合一”，在全省率先推出电子营业执照，五年来净增市场主体12.1万家。建立全省首个综合性网上“银税互动”平台，累计为小微企业发放贷款33亿元。农村集体经济“三资”监管走在全省前列。建成投资贸易双平台，设立“走出去”综合服务中心和采购服务中心。中山港口岸扩大开放通过国家验收。实现与澳门游艇自由行。粤澳全面合作示范区获国家支持，落实粤港、粤澳框架协议考核居珠三角前列。

　　过去五年，是基础建设快速推进的五年。城市总规（2010-2020年）获国家部际联席会议通过，建立“三规合一”协调机制。五年累计投入250亿元推动交通大发展，全市公路通车里程达3235公里，每百平方公里公路密度由2011年的106公里提高到180公里。深中通道动工。广珠西线、广中江高速一期、翠亨快线、三角快线、沙古公路、105国道北段、阜港公路、中山港新桥等建成通车，古神公路二期北段、城桂路中心城区段等完成改造。S111、S365 中山段升级为228国道。群众出行“最后一公里”工程全面完成，累计改造和建设村级公路608公里。五年来改造危桥343座，完成主干农路硬底化888公里。黄圃港开港运营。能源、信息、环保等基础设施加快推进。光纤入户率达90%，连续四年居全省第1。五年来改造“三旧”项目197个，处置闲置土地5.7万亩。古镇入选国家住建部首批“中国特色小镇”。

　　（上接1版）

　　过去五年，是生态环境得到提升的五年。成为国家生态市。五年累计投入51.3亿元用于绿化，建成大尖山森林公园等25个森林公园和湿地公园，完成和提升道路绿化1100多公里，全市森林覆盖率达23.06%。成功创建广东名村12个、生态示范村247个，秀美村庄创建实现全覆盖。五年来淘汰黄标车和老旧车超10万辆。空气质量保持在全国前列。完成中心城区雨污分流工程。南粤水更清行动综合得分连续三年居全省第1。单位生产总值能耗累计下降17.5%，主要污染物总量减排考核连续四年被评为优秀。

　　过去五年，是民生福祉持续改善的五年。财政民生支出逐年增长，平均占财政支出比重近七成。每年十件民生实事均顺利完成。2015年居民人均可支配收入达35712元，是2011年的1.5倍。城乡居民收入差距全省最小。五年来城镇新增就业27.1万人，城镇登记失业率保持在2.3%以下。社会保障体系不断健全，城镇职工社会保险参保超千万人次，企业职工基本养老金提高到每月人均2494元，低保、低收入标准分别提高到629元和943元。重特大疾病医疗救助实现户籍居民全覆盖。五年累计投入13.1亿元，建成保障性住房1.26万套，完成棚户区改造2846户，发放租赁补贴2641户。五年累计投入462.5亿元发展教育，公益普惠性幼儿园比例达79.4%，成为全国义务教育发展基本均衡市和全省推进教育现代化先进市，2016年高考本科、重点本科和总上线率均居全省第1，高中阶段学校实现市级统筹办学，中山职院、火炬职院进入省首批一流高职院校建设名录。全民健身事业蓬勃发展，成功举办"逸仙杯"国际马拉松赛等一批重要体育活动。公立医院综合改革稳步推进，实现检查结果互认、诊疗信息互联互通，分级诊疗、"医联体"、家庭医生式服务模式得到上级充分肯定，2016年新增民营医疗机构98家。蝉联国家卫生城市，建成国家级卫生镇18个。全面两孩政策稳步落实。人均预期寿命提高到79.43岁。建成市、镇、市场三级食品药品检测体系，市食品药品检验检测中心投入使用。

　　过去五年，是社会建设不断进步的五年。蝉联全国文明城市称号。孙中山故里旅游区成为国家5A 级旅游景区，成功举办孙中山诞辰150周年纪念活动，承办两届海峡两岸中山论坛。蝉联全国创新社会治理优秀城市。积分制获全国地方政府创新奖，近8.2万名流动人员及其子女获得积分入户入学入住公租房资格。建成286个社区综合服务大厅和82个行政村（社区）综合性文化服务中心。农村社区建设试点有序推进，村（居）委会特别委员、监督委员会建设得到省的肯定。信访秩序持续好转，"诉访分离"效果明显。社矫安帮服务中心实现镇区全覆盖。实施"全民治安"工程，第五次获评全国社会治安综合治理优秀地市，第三次捧得"长安杯"。深入推进"飓风2016"等系列专项行动，重拳打击突出刑事犯罪，2016年刑事发案下降12.2%，电信诈骗案件下降超3成。连续三年在省消防考核中被评为优秀。连续两次荣膺全国双拥模范城称号。外事侨务工作获外交部 "服务国内发展突出贡献奖"。扶贫"双到"成效显著，新时期精准扶贫工作开局良好，对口帮扶潮州、肇庆和支援云南昭通、西藏工布江达等工作有序开展。检验检疫、安全生产、应急管理、民族宗教、统计、审计、国防建设、海洋渔业、老龄、妇女儿童、残联、红十字会、气象、人防、档案、方志等各项工作取得新成绩。

　　过去五年，我们不忘初心，砥砺前行，始终注重加强政府自身建设，提高政府的执行力和公信力。深入开展党的群众路线教育实践活动、"三严三实"专题教育和"两学一做"学习教育，更加坚定广大党员干部的理想信念，不断提高干部队伍的思想觉悟和工作水平。全面落实市政府党组主体责任，出台实施市政府党组全面从严治党相关制度。认真落实市人大及其常委会各项决议决定，主动加强同人民政协联系，五年来办理大会议案4件、人大代表建议665件和政协提案1148件，建议提案办理工作成效显著。推进阳光政府建设，进一步深化政务公开、网络问政，充分听取社会各界的意见建议。地方立法有序开展。建成市、镇、村三级公共法律服务实体和24小时线上法律服务平台。稳步推进法治政府建设，率先在全省建立行政执法全过程网上记录和行政执法信息化机制，行政处罚自由裁量权标准化管理改革经验在全国推广，在全省依法行政考核中被评为优秀。深入推进"督考合一"政府绩效管理模式，提升政务工作实效。积极构建新型政商关系。严格执行中央八项规定，强化"三公"经费管理，加强对公务消费开支的监督检查，稳妥推进公务用车制度改革。加强廉政建设，强化监察审计等监督，推进对公共资金、国有资产、国有资源和领导干部履行经济责任情况审计监督全覆盖。

　　各位代表！发展来自于真抓实干，成绩得力于凝心聚力。回顾过去五年，我们深深地感到，成绩来之不易，经验弥足珍贵。这些成绩的取得，是省委、省政府和市委正确领导的结果，是市人大及其常委会和市政协监督支持的结果，是全市干部群众团结奋斗的结果，是海内外各界关心帮助的结果。在此，我代表市政府，向全市广大工人、农民、知识分子、企业家、干部群众，向各民主党派、工商联、各人民团体、无党派人士和社会各界人士，向驻我市解放军和武警官兵、人民警察，向所有关心支持中山发展的老领导、老同志、港澳台同胞、海外侨胞和国内外友人表示衷心感谢！

　　在看到成绩的同时，我们也清醒地认识到，当前我市正处在转型发展的关键时期，仍然面临着不少困难和问题，突出表现在：传统动能乏力，新的增长点尚在培育；社会创新活力不足，企业自主创新能力不强；人才结构不够合理，创新型、实用型人才培养和引进力度有待加大；营商环境有待改善，市场活力没有充分释放；重大项目不多，土地碎片化和利用效率不高等问题依然存在；城市建设和管理水平有待提高，交通拥堵、水环境污染等"城市病"治理任重道远；在医疗、教育等领域仍然存在不少短板，改善民生还须付出更大努力；部分行政人员担当意识不强，行政效能与群众期待还有一定差距等。对此，我们将高度重视，采取有力措施认真加以解决。

　　今后五年展望

　　各位代表！今后五年，是中山发展的关键时期。综观国内外形势，世界经济在深度调整中曲折复苏，全国全省经济发展长期向好的基本面没有发生根本性变化。随着粤港澳大湾区、珠三角国家自主创新示范区的加快建设，以及区域重大交通基础设施的集结成网，中山的区位优势得到前所未有的突显。同时，广东省 "十三五"规划纲要赋予中山"四个定位"，即把中山建设成为世界级现代装备制造业基地、珠江西岸区域性综合交通枢纽、区域科技创新研发中心和珠三角宜居精品城市。这是省委、省政府为中山确定的战略目标，开启了中山发展新征程。刚刚过去的一年，在中山改革发展的关键时刻，省委、省政府主要领导多次亲临视察并发表重要讲话，就中山交通建设、产业发展、科技创新、空间布局、生态建设和社会治理等，作出了重要部署，为中山发展指明了方向。我们要坚决贯彻省委、省政府的决策部署，增强战略意识、机遇意识和争先意识，坚持目标导向和问题导向，以更加开阔的视野、更加主动的姿态、更加有力的举措，锐意创新、团结奋进，着力构筑体制、政策、人才、环境、服务等新的比较优势，奋力推动中山新一轮跨越发展。

　　今后五年工作的指导思想是：高举中国特色社会主义伟大旗帜，以马列主义、毛泽东思想、邓小平理论、"三个代表"重要思想、科学发展观为指导，深入学习贯彻习近平总书记系列重要讲话精神，认真贯彻省委十一届八次全会和市第十四次党代会工作部署，统筹推进"五位一体"总体布局和协调推进"四个全面"战略布局，践行创新、协调、绿色、开放、共享的发展理念，适应把握引领经济发展新常态，进一步加强党的领导，强化统筹协调发展，大力实施供给侧结构性改革根本性战略和创新驱动发展核心战略，努力建设充满创新活力的和美宜居城市，率先全面建成小康社会，向基本实现社会主义现代化迈出坚实步伐。

　　今后五年，我们要牢牢把握省委、省政府赋予中山的"四个定位"要求，坚持创新驱动，集聚创新创业要素引领发展；持续提升城市宜居环境，建设健康城市。营造整洁、畅顺、绿色、安全的城市环境；营造市场化、法治化、国际化的营商环境；营造风清气正、干事创业的政治生态环境。到2020年，力争实现生产总值年均增长8.5%左右，先进制造业占制造业增加值比重达50%，全社会研发经费占生产总值比重达2.9%。

　　2017年工作安排

　　各位代表！2017年是供给侧结构性改革的深化之年，也是我市率先全面建成小康社会的攻坚之年。做好今年工作意义重大。我们将牢记责任和使命，凝心聚力、励精图治，全力赢得新一轮发展主动权。

　　2017 年经济社会发展的主要预期目标是：生产总值增长8%以上，固定资产投资增长13%，社会消费品零售总额增长10%，进出口总额和出口总额增长1%，地方一般公共预算收入增长8%，居民消费价格涨幅控制在3%左右，居民人均可支配收入增长高于经济增长，城镇登记失业率控制在3.5%以内，节能减排降碳约束性指标达到省下达 "十三五"总目标进度要求。

　　2017年，我们将坚持稳中求进工作总基调，紧紧围绕"四个定位"战略目标，以提高发展质量和效益为中心，以推进供给侧结构性改革为主线，以构筑比较优势为重点，深化创新驱动，加快转型升级，推动组团发展，打造交通枢纽，改进民生福祉，不断提高城市核心竞争力。

　　为实现上述目标，我们将做好以下工作。

　　●一、着力促进平稳增长，推动经济发展稳中有进

　　坚持将稳增长摆在更加突出的位置，把握稳增长、调结构、塑优势的平衡，采取有力举措对冲下行压力，努力保持经济中高速增长。

　　打造重大产业平台。坚持以大平台带动大发展，统筹全市资源，在优化提升44个市级工业园区的基础上，在各个组团内谋划建设重大产业平台，重点发展高技术产业平台（火炬开发区、翠亨新区)、先进制造业平台（民众、三角）、智能制造装备产业平台（板芙）、精密制造业平台（坦洲）、现代物流业发展平台（黄圃）、传统产业转型升级平台（小榄、古镇）等一批重大产业平台。在用地规模、财政资金、社会融资、项目引进等方面向重大产业平台倾斜。加大招商引资力度，发挥行业协会、海外港澳乡亲和各类专业招商公司作用，瞄准国内外知名大企业，开展定向招商、委托招商和以商引商，着力引进一批优质大项目。

　　大力振兴实体经济。坚持制造业立市不动摇，发展高端、高质、高新制造业，打造"智"造强市。促进各方面资源要素加快向实体经济集聚，发挥产业扶持资金杠杆效应，引导社会资本重点投向先进装备、电子信息、生物医药、新能源、新材料、家用电器、五金制品、智能家居等产业。积极参与珠江西岸先进装备制造产业带建设，重点发展"三有"工作母机制造业。加快推进哈工大无人装备产业基地、大洋电机新能源汽车动力总成及氢能源电池模组、明阳风电碲化镉和生波尔CIGS第三代太阳能薄膜电池等项目落地。推动传统产业向提品质、树品牌方向加快转型，落实智能制造规划，加快建设智能制造公共服务平台，引导灯饰、服装、红木等传统优势产业产品智能化改造。鼓励企业增资扩产，引导550家规模以上工业企业实施技术改造。落实支持民营经济发展的政策措施，推动民营企业提质增效。建立大型骨干企业培育库，做强做优龙头骨干企业。大力培育高成长性的中小企业，促进小微企业上规上限。优先发展现代服务业，推进"十三五"国家服务业综合改革试点建设，加快现代服务业集聚区建设，规划建设一批生产性服务业示范区，新增100家规上限上服务业企业。创建全国质量强市示范城市，提升产品、工程、服务、环境质量，塑造质量优势。培育和引进智能化服务、检验检测、第三方认证、品牌创建、知识产权保护等方面的机构，创建3家省级以上公共检测技术服务平台。

　　积极扩大有效投资。发挥投资的关键作用，使之成为稳增长、调结构、促转型的"顶梁柱"。突出抓好重点领域投资，大规模增加基础设施投入，加快轨道及高快速交通网、信息网络、环保水利、市政能源等基础设施项目建设，通过政府投资带动社会资本投向基础设施领域。突出抓好总投资达3654 亿元的160个市重点项目建设，完成年度市重点项目投资480亿元。突出抓好亿元以上非房地产项目建设，推进一批先进制造业和现代服务业项目建设。推动政府与社会资本合作，落实支持民间投资各项政策，加快建立市场准入负面清单制度，激发民间投资活力。

　　深入挖掘消费潜力。继续开展各大节日惠民促销活动。引导生活服务业向精细化、高品质转变，培育发展信息消费、文化教育、体育健身、游艇旅游等新兴消费热点。推动中山货全国行。实施 "互联网+流通"计划，推动商贸企业发展线上线下业务，支持电子商务与物流快递协同发展。办好古镇灯博会、港口游博会、火炬区装备展等系列活动。

　　扎实推进外贸企稳向好。加大出口信保支持力度，引导企业培育出口品牌和建设海外营销网络，开拓东盟、中东、非洲等"一带一路"沿线国家和新兴市场。推进国际贸易"单一窗口"平台建设，加快"三互"大通关改革，降低企业通关成本，提高通关效率。支持外贸综合服务企业发展，推进跨境电商平台建设，争取成为国家市场采购试点。加大进口支持力度。

　　加快转变农业发展方式。以"两岸四地"现代农业合作示范区建设为抓手，建设精致农业示范园。加快建设一批设施农业示范基地，推动互联网与现代农业深度融合发展。创建一批农业公园和农业综合体，促进农业与生态休闲旅游融合发展。推进农业"三项补贴"改革。推行农业标准化生产。深化农村集体产权制度改革，推进农村土地承包经营权确权登记颁证工作。综合利用海洋资源，鼓励渔民转产转业。

　　●二、着力实施创新驱动，提升经济发展质量效益

　　坚持把科技创新作为推动转型升级的最有力抓手，以产业发展牵引科技创新，以科技创新支撑产业发展，营造创新和人才优势，打造区域科技创新研发中心。

　　加快珠三角国家自主创新示范区建设。编制我市自创区发展规划，落实国家自创区"6+4"自主创新政策，研究制定支持自创区发展的政策措施。支持翠亨新区与中科院、哈工大合作，推动科技成果转化。建设健康科技、光成像与光电子信息等一批创新型产业集群，提升火炬开发区、翠亨新区产业集聚水平。加强与周边自贸区的交流合作。

　　加大科技创新力度。围绕互联网+、智能制造、新材料等产业，打造一批创新载体，建设20个新型研发机构。加大科技公共服务平台建设力度，推动科技服务集聚基地建设。加大科技创新券、企业研发费补助等政策扶持力度，支持科技型企业发展。新增高新技术培育入库企业200家以上。加强关键核心技术攻关，实施一批重大科技项目。

　　培育壮大新动能。大力发展"四新"经济（新技术、新产业、新业态、新商业模式），催生新的经济增长点。扶持先进装备制造、健康科技、文化旅游等产业发展，加快建设中德 （中山）生物医药产业园、中瑞（欧）工业园。实施互联网+和大数据行动计划，推动互联网+在经济、社会、民生等领域的应用，推进中山智能制造大数据产业园和全通星海大数据孵化园建设。培育发展电子商务服务产业，建设电子商务集聚区。

　　推动科技金融融合发展。构建以财政资金为引导，银行、保险、风投、创投等各类资本参与的科技金融生态圈。鼓励设立天使基金、科技成果转化基金。充分发挥科技信贷风险准备金池的作用，加快发展科技信贷。推进知识产权质押融资，促进专利技术产业化。探索 "科技银行+投资机构"的联动模式。推进融资租赁、融资担保、科技保险等金融机构发展。搭建科技型中小企业投融资服务平台。举办创新创业大赛。深化建设众创金融街。

　　实施更加开放的人才政策。优化人才引进政策，推出更有吸引力的人才措施。发挥企业作为吸引人才的主体作用，打造一批集聚人才的高端载体和平台。吸引高端人才和创新项目进驻。加大创新型领军人才和团队的扶持力度。大力培养应用型技能人才。提高人才服务效率和质量。支持企业通过股权、期权、分红等方式激励优秀人才。

　　●三、着力加强建设管理，积极打造宜居精品城市

　　坚持规划引领，强化战略思维和全局视野，加强城市空间战略研究，立足高起点规划、高标准建设、高水平管理、高品质配套，促进生产空间集约高效、生活空间宜居舒适、生态空间山清水秀。

　　实施组团式发展战略。根据空间布局和产业特征，构建"一中心、四组团"发展格局。强化功能定位和权限匹配，推动各组团差异化发展。优化各种资源配置，建立适应组团发展的治理体制和体系。实施 "强心"战略，提高中心城区首位度。加强中心城区与东部组团、东北组团、西北组团、南部组团的联系，打造中心至组团的高快速路网。加强各组团与周边城市的对接合作，动工建设一批市际跨界道路。加强组团之间的联系。

　　全力打好交通攻坚战。加快轨道交通建设，配合推进深茂铁路、南沙港铁路建设，谋划广州直通中山的城市地铁，启动城市轨道1号线一期工程，加快对接国家高铁网，实现从珠海开出的所有长途跨线动车组列车均在中山站停车办理客运业务，开通直达北京、上海、长沙、石家庄、深圳、厦门、杭州、宁波、贵阳、桂林等城市的高铁服务。依托现有站点，建设综合轨道交通枢纽站。加快高速公路建设，推进中开高速和小榄支线、南中高速、东部外环高速、西部外环高速建设，配合推进深中通道、广中江高速、香海高速建设，打造贯穿市域的高速公路网。加快干线公路和市政道路快速化改造，推进105国道南段、长江路、博爱路2个交叉路口、东二环等改造，启动南三公路、西二环、马石路、纵四线终点段等建设，推进康华路、北二环、南朗快线、黄圃快线、大南公路、岭栏路、西北三环等建设。加快畅通城市交通微循环，落实《主城区近期交通改善详细规划》，加快孙文东路、景观路等一批道路的精细化改造，打通大沙南路、富康北路等5条断头路，启动东华路、齐学路等5条断头路建设。创建省级公交示范城市，优化公交线网，加快改造市汽车总站等枢纽站场。加快"一港五区"建设，以国家验收对外开放口岸为契机，加快码头泊位及配套设施建设，推进航道改造升级，提高货物吞吐量。以黄圃港区为依托，连接南沙港铁路黄圃货运站，打造连接欧洲快线的水铁联运、公铁联运物流中心。以神湾港区为依托，打造木材、水果等商品综合进出口基地。加快中山港客运码头东移，谋划开通与深圳机场和前海的客运海上穿梭巴士。

　　塑造城市品质风貌。推进广东省新型城镇化 "2511"综合试点工作。注重城市设计的整体性，加强重要景观景点、廊道的空间设计。加强对老城区的风貌保护，完成环铁城步道改造。挖掘地域文化特色，保护传承和合理利用文化遗产。推进岐澳古道保护开发。实施东区中心商务区整体形象改造工程。推进岐江新城区域规划和建设。启动翠亨新区滨河整治水利工程建设，推进坦洲海堤加固一期等工程建设。加强城市立面管理，实施城市亮化工程，提升城市魅力。

　　强化集约节约用地。完善"三规合一"成果，推动多规融合。争取市土规、城规方案尽快获国家批准，完成各镇总规编制。加快地籍调查和不动产登记，建立不动产登记信息管理基础平台。重点推进10个连片"三旧"改造试点项目，有序推进城市功能修补和有机更新。加快批而未供用地供应，处置闲置用地5000亩，加快"锌铁棚"等低效用地的盘活利用。加强土地整治，逐步妥善解决历史遗留问题。

　　建设特色小镇。将特色小镇打造成为转型升级的新载体，推进"产、城、人、文"有机结合。按照产镇融合模式，推进古镇灯饰小镇等一批特色小镇建设。开展美丽城乡创建行动，改善农村人居环境，打造秀美村庄"升级版"。发挥生态、农业、休闲、人文、工业等方面的优势，推进全域旅游，提升旅游服务质量，争创国家全域旅游示范区。完善孙中山故里旅游景区。

　　优化城市管理。坚持科学化、精细化、品质化管理，提高城市管理水平。完成城市管理体制改革，理顺城市管理和执法机制，构建"大城管"格局。加快城市管理和服务体系智能化建设，完善数字城管系统。开展交通综合治理，运用大数据优化智能交通管理。完善慢行交通设施。创新城市维护管理，推进市政基础设施、市政公用事业、公共交通、便民服务等设施的市场化运营。加快市政、环卫、园林项目建设。开展"四看"市容环境综合整治行动。实施干线公路路域环境综合治理。加强施工管理，减少对市民工作和生活的影响。

　　●四、着力深化改革开放，激发经济社会发展活力

　　坚持向改革要红利、以开放赢空间，紧紧扭住关键性改革不放松，持续推动深层次开放不停步，努力营造市场化、法治化、国际化营商环境。

　　深入推进供给侧结构性改革。坚持优化存量、引导增量与主动减量相结合，注重精准施策，提高供给体系的质量和效率。打好"去降补"组合拳，稳妥推进僵尸企业处置，支持企业兼并重组、债务重组或破产清算。加快建立专业化住房租赁平台，培育住房租赁市场。统筹房地产源头供应管理，推进商品房去库存，促进房地产市场平稳健康发展。防范和处置金融风险，确保金融机构杠杆率控制在合理水平。开展降本增效专项行动，落实国家减税降费政策，实施涉企收费目录清单管理，取消车辆通行费年票制。加快补齐交通、能源、环保、信息基础设施等重点领域短板。深化市属国有企业、镇（区）属公有企业改革。

　　加大"放管服"改革力度。加快推进项目投资高效审批改革。实行企业投资项目清单管理，最大限度缩减投资项目审批事项。开展"互联网+政务导办"，实行重点项目导办代办和绿色通道服务。深化 "一门式、一网式"政府服务模式改革，加快基层公共服务综合平台建设，完善"市、镇、村、家"四级政务服务体系。深化商事制度改革，根据省部署推进工商登记全程电子化。合理划分市镇事权，推进镇区行政管理体制改革。支持火炬开发区、翠亨新区开展体制机制创新试点。

　　深化财税金融领域改革。推进全口径预算管理和项目库管理改革，强化预算执行管理。加大转移支付力度，完善财政管理体制。深化税制改革，健全地方税费收入体系。发挥财政资金引导作用，鼓励金融资源支持实体经济发展。加强小额贷款公司监管，支持融资担保业发展。支持企业上市、新三板挂牌，鼓励企业定向增发及通过企业债、公司债、私募债等形式直接融资。拓展农业"政银保"项目，发展适合农户需求的小额信贷产品。探索开展老年人住房反向抵押养老保险试点。

　　推进"信用中山"建设。积极构建"政府+企业+重点人群"的社会信用体系。加快政务诚信建设，落实行政许可和行政处罚信用信息公示制度。完善公共信用信息管理系统。深化商务诚信建设，落实诚信红黑榜公布制度，构建守信激励、失信惩戒机制。加强信用服务市场建设，支持本土信用服务机构发展壮大。倡导诚实守信风尚。

　　高水平扩大对外开放。实施参与"一带一路"行动计划。复制推广自贸区政策。推动中山保税物流中心争创综合保税区。发挥口岸扩大开放及游艇自由行平台作用，助推临海制造、船舶维修、游艇等产业发展。严格执行国家放宽外商投资准入政策。落实粤港、粤澳合作框架协议，主动融入粤港澳大湾区建设。抓好珠三角"九年大跨越"收官工作。发挥重点侨乡资源优势和友城、友协等平台作用，扩大国际交流合作。

　　●五、着力保护生态环境，构建绿色城市发展格局

　　坚持绿色发展理念，树立"绿水青山就是金山银山"的强烈意识，改善生态环境质量，筑牢环境优势，推动人与自然和谐发展，营造天蓝、地绿、水净的城市环境。

　　创造更多绿色空间。开展"三年绿化美化"提升工程，提高市域森林覆盖率。创建国家森林城市，争创五桂山国家级自然保护区、金钟湖国家级森林公园、翠亨新区国家级湿地公园，建设南朗崖口、坦洲金斗湾等省级湿地公园，打造一批市级镇级湿地公园。推进古香林风景名胜区建设，加快金钟湖公园、古香林公园、金字山公园、气象公园、儿童公园、新安公园（环保教育基地）、树木园三期等城市公园建设。推进干线公路绿化美化升级改造工程。加快岐江河沿江公共绿地和景观带建设。启动森林小镇建设，打造森林生态廊道。推动全民绿化，引导社会力量投资造林。

　　加强环境保护治理。创建国家生态文明建设示范市。严格划定生态保护红线，健全生态补偿动态调整机制。开展生态修复，恢复城市自然生态。加强重点排污企业、重点污染源和减排项目监管。建立市镇两级河长体系。推进岐江河流域综合治理，加大黑臭水体整治力度。抓好大气污染防治，严控建筑施工扬尘，力争PM2.5年均浓度控制在35微克/立方米以内、空气质量优良天数达90%以上。推进土壤污染防治。加强海域环境监控，保护海洋生态环境。加快垃圾处理设施建设，推进中心组团垃圾综合处理基地三期、北部组团垃圾综合处理基地三期建设，实现南部组团垃圾综合处理基地焚烧发电厂投入运行。加大环境监管执法力度，实施跨镇区环保联合执法。落实中央环保督察整改要求，解决涉及生态环境保护的突出问题。

　　推进节能减排降碳。开展碳普惠制试点建设。发展节能环保产业，提高项目准入门槛。鼓励节能技术应用，推动电机能效提升，支持企业清洁生产。推广新能源汽车，淘汰黄标车和老旧车辆。推进建筑节能与绿色建筑。建设重点用能企业能管中心，推动企业节电和电能替代5000万千瓦时。落实国家和省电力体制改革部署。

　　（上接2版）

　　●六、着力加强社会建设，持续构建和谐善治中山

　　坚持依法治理、共同治理，按照人人参与、人人尽力、人人享有的要求，扩大公共产品和公共服务供给，维护社会公平正义，促进社会和谐进步。

　　加强社会治理创新。推进全民参与社会治理，推动中国社会创新（中山）基地和全民公益园建设，打造一批社会服务综合平台。依法开展村级组织换届，推进村（居）"政经分离"改革。深化"专业社工、全民义工"试点，培育和规范社会组织发展。落实户籍制度改革政策，完善积分制管理制度，拓展居住证社会应用功能，发展异地务工人员服务组织。促进非户籍人口参与基层治理。加强政策引导，支持住宅小区成立业主委员会。健全公共法律服务体系。加强摩托车、电动自行车规范管理。促进网约车有序发展。

　　深化平安中山建设。推进"智慧公安"建设，提升打击突出违法犯罪水平，构建立体化治安防控体系。整合、改造公安反恐指挥中心，做好防恐防暴工作。加强互联网安全管理，严打网络违法犯罪。深化 "平安细胞"创建，实施出租屋安居环境提升计划，落实出租屋"门禁+视频"建设。健全社会稳定风险评估机制，强化矛盾纠纷排查化解。做好综治信访维稳工作，加强信访法治化建设。推进特种设备安全监管体制改革，加强特种设备运行安全监管。落实安全生产"一岗双责"，强化企业的主体责任，加大安全生产监管力度，加强隐患排查和事故防控。做好应急管理工作。加快水利设施建设，推进气象现代化，完善防灾减灾救灾体系。

　　增强文化整体实力。深化文明城市创建成果，提高市民文明素质和城市文明程度。加强社会主义核心价值观教育。推进公共文化服务标准化、均等化、数字化和社会化建设，60%以上的行政村（社区）建成基层综合性文化服务中心。实施文化惠民工程，倡导全民阅读，加快中山纪念图书馆建设，构建图书馆总分馆体系，实现市镇两级图书通借通还。推进中山博物馆群建设。加强文化遗产保护利用，完成孙中山故居纪念馆陈列展览升级，推进翠亨旧村"活化"和全面开放。鼓励文艺创作，营造有利于出人才、出精品的良好环境。推动文化创意与制造业融合发展，做大做强文化产业。

　　发展各项社会事业。实施精准扶贫精准脱贫，做好对口帮扶和支援工作。做好市内帮扶工作，支持困难村解困发展。加强国防建设，扎实开展双拥工作，认真落实优抚安置政策。健全家庭服务体系。开展妇婴关爱行动。完成第二次全国地名普查补查工作。做好检验检疫、台务、民族宗教、妇女儿童、统计、审计、档案、方志等工作。

　　●七、着力保障改善民生，提高人民群众幸福指数

　　坚持把改进民生放在重中之重的位置，积极回应群众期待，大力解决民生突出问题，补短板、兜底线、促提升，扩大群众的获得感，让全市人民共建共享改革发展成果。

　　完善社会保障体系。安排8.46亿元用于医疗保障、生活保障等社会保障支出。修订基本医疗及大病医疗保险办法，促进养老金正常增长。推进机关事业单位养老保险制度改革，推动实行企业（职业）年金制度，建立多层次社会养老保险体系。推动更高质量就业，启动"易创空间"创业孵化基地建设，加大重点群体就业创业帮扶。健全职工工资稳定增长机制，促进城乡居民收入持续增加。提高低保标准和补助水平，完善社会救助制度。采取实物配租与货币补贴相结合的方式，加大住房货币化保障力度。推进养老机构社会化改革，加快居家养老服务标准化建设。完善无障碍公共服务设施。做好慈善、红十字会工作。

　　提供更优质的教育。发展公益普惠性幼儿园，增加规范化幼儿园学位。大力推动公办中小学学校建设，新建、改建、扩建公办学校33所。深化高中招生制度改革，全面提高教育质量。深化职业教育综合改革，探索中高职一体化办学，建设中德合作职业技术人才培训中心。加快高水平大学建设，推进中外合作办学。扶持和规范民办教育发展。

　　加快建设健康中山。树立"大健康"理念，加快卫生强市建设，落实"强基层、补短板、优供给"三年行动计划。深化公立医院改革，推动医疗、医保、医药联动。加大医疗卫生人才培养和引进力度。加快分级诊疗体系建设。鼓励社会办医，提升基层中医药服务能力，探索医养结合服务模式。加快区域卫生信息平台建设。筹建新的市人民医院，谋划建设市儿童医院。实施出生缺陷综合防控项目。加强疾病防控体系建设，提高突发公共卫生应急能力。优化健康服务。发展健康产业。办好中山健康论坛和吴阶平医学奖颁奖大会。加快计生转型发展。创建食品安全示范城市，强化食品药品生产经营全程监管，完善食品药品检验检测体系。广泛开展全民健身活动，以组团布局为原则新建4个体育公园，更新改造老旧体育设施，加快发展体育产业。

　　办好十件民生实事。坚持按照"市民点菜、政府买单"的思路，从群众的实际需求出发，继续集中力量办好十件民生实事。一是优化交通管理；二是创建食品安全示范城市；三是加强打击和防范盗抢骗；四是提升公交服务；五是整治黑臭水体；六是加强施工管理；七是优化政务服务；八是完善儿童活动设施；九是完善社保服务设施；十是推行公安便民服务。

　　●八、着力加强自身建设，打造法治高效廉洁政府

　　坚持从严治政，切实增强政治意识、大局意识、核心意识、看齐意识，坚决维护以习近平同志为核心的党中央权威，勤政为民，开拓进取，不断提高政府治理现代化水平。

　　全面推进依法行政。坚持用法治思维和法治方式履行职能、调节经济、推动改革。推进法治政府示范区建设，探索行政复议快速立案机制；建立行政调解制度；构建政府立法的公众参与机制；完善行政应诉制度；严格落实重大行政决策合法性审查制度，切实将政府行为纳入法治轨道。依法及时向市人大及其常委会报告工作，认真执行市人大及其常委会的各项决议决定，积极支持人民政协履行政治协商、民主监督、参政议政职能，做好人大代表建议和政协提案办理工作，提高质量和成效，自觉接受监督。

　　全面推进政务公开。坚持以公开为常态、不公开为例外，推进决策、执行、管理、服务、结果"五公开"。深化细化财政预决算、减税降费、公共资源配置、重大项目建设、就业创业、公益事业、公共安全、教育环保等重点领域的信息公开。建设好政府门户网站和 "中山发布"，建立常态性的新闻发言人制度，畅通政府与市民的沟通渠道。引导形成良好的社会预期。推进智库建设，吸纳社会和民间智慧。深化省网上办事大厅中山分厅建设，发挥市长信箱、政务服务热线、网络问政平台等政民互动平台作用，自觉接受舆论监督和公众监督。

　　全面推进作风建设。坚定理想信念，严守政治纪律和政治规矩。强化服务基层、服务企业、服务群众的意识，不断提高政府服务的质量。强化担当精神，鼓励主动作为，营造想干事、能干事、干成事的良好氛围。建立健全廉政建设与业务工作融合机制，完善行政权力内部流程控制制度。加强干部队伍作风建设，贯彻落实《中国共产党问责条例》、《中国共产党党内监督条例》、《中国共产党廉洁自律准则》，深化"为官不为"专项整治，加大监督问责力度。深化绩效管理，强化督查督办，确保各项目标任务落到实处。加大审计改革力度，依法开展审计监督。建立健全预防腐败清单制度，坚持无禁区、全覆盖、零容忍惩治腐败。严格落实中央八项规定，加强"三公"经费管理。

　　各位代表，新的征程充满希望，新的梦想扬帆起航。让我们在中共中山市委的坚强领导下，凝心聚力，奋勇拼搏，用激情绘就蓝图，用实干成就梦想，为率先全面建成小康社会、建设充满创新活力的和美宜居城市而努力奋斗！
